# The efficacy and safety of combined 0.01% atropine and orthokeratology for childhood myopia control: a 2-year randomized clinical trial

**DOI:** 10.3389/fped.2026.1809296

**Published:** 2026-04-30

**Authors:** Ying Yuan, Yuqi Deng, Chengcheng Zhu, Xinting Liu, Wei Zhang, Bingru Zheng, Xinjie Mao, Xiao Yang, Bilian Ke

**Affiliations:** 1 Department of Ophthalmology, Shanghai General Hospital, Shanghai Jiao Tong University School of Medicine, Shanghai, China; 2 National Clinical Research Center for Eye Diseases, Shanghai, China; 3 Department of Ophthalmology, Ren Ji Hospital, Shanghai Jiao Tong University School of Medicine, Shanghai, China; 4 Department of Ophthalmology, Qilu Hospital of Shandong University Dezhou Hospital, Dezhou, Shandong, China; 5 National Clinical Research Center for Ocular Diseases, Eye Hospital, Wenzhou Medical University, Wenzhou, China; 6 Biomedical Informatics & Statistics Center, School of Public Health, Fudan University, Shanghai, China; 7 Shenzhen Eye Hospital, Jinan University, Shenzhen Eye Institute, Shenzhen, China; 8 State Key Laboratory of Ophthalmology, Zhongshan Ophthalmic Center Sun Yat-Sen University, Guangzhou, China

**Keywords:** 0.01% atropine, childhood myopia, multicenter trial, myopia control, orthokeratology

## Abstract

**Purpose:**

To explore the efficacy and safety of combined low-concentration atropine and orthokeratology lenses (AOK) for slowing the progression of myopia.

**Methods:**

In this randomized, placebo-controlled, double-blinded, multicenter clinical trial. Children (*n* = 96) aged 8–12 years with myopia between −1.00D and −4.00D and astigmatism of no more than 1.5D in either eye were randomly assigned to the AOK or orthokeratology (OK) group. The primary outcome, axial elongation, was examined at baseline and visited at 6, 12, 18 and 24 months, along with secondary outcomes, including tear meniscus height (TMH), non-invasive break-up time (NIBUT), visual acuity and dynamic changes of pupillary light reflex.

**Results:**

Over the 24 months, the axial elongation was significantly slower in the AOK group than in the OK group (0.33 ± 0.17 mm vs. 0.43 ± 0.16 mm, respectively; *p* = 0.004). In the subgroup analysis, there were significant differences in the change of axial length in the subgroup of subjects with an initial spherical equivalent refraction (SER) of −1.00 to −3.00D or aged 8–10 years (*p* = 0.017, 0.021, respectively). At the 24-month visit, the changes in TMH, NIBUT, visual acuity and pupil size in the AOK group were insignificantly different from those in the OK group (p>0.05, respectively). The latency of the pupillary light reflex was significantly longer in the AOK group than in the OK group (*p* = 0.029).

**Conclusion:**

The combination of 0.01% atropine and orthokeratology produced a combined effect in slowing axial elongation over 2 years, with good tolerability and minimal ocular surface impact.

**Clinical Trial Registration:**

https://www.chictr.org.cn/showprojEN.html?proj=29216, identifier ChiCTR1800018419.

## 1 Introduction

The prevalence of myopia has been increasing worldwide and has become a major public health concern in recent decades, especially in East Asia (1). Myopia progression is usually characterized by axial elongation, which increases the risk of developing sight-threatening ocular complications, including myopic maculopathy, cataract and glaucoma (2). Therefore, it is vital to explore effective strategies to slow myopia progression.

Atropine and orthokeratology were proposed to be the most effective methods of slowing myopia progression in children (3). Atropine, a nonselective anticholinergic agent, has been widely used to control myopia progression. The Atropine for the Treatment of Childhood Myopia (ATOM) studies revealed that atropine has a dose-dependent clinical efficacy in slowing myopia progression (4, 5). In the ATOM2 study, when comparing 0.5%, 0.1%, and 0.01% atropine, 0.01% atropine had a similar effect but fewer adverse vision-related events (4, 6). However, atropine ophthalmic solution treatment must be combined with spectacles, contact lenses or orthokeratology (OK) lenses to provide refractive correction. OK lenses were specially designed to temporarily reduce myopia severity by reshaping the cornea. It has also been suggested that OK lenses could inhibit myopia progression by reducing peripheral hyperopic defocus (7, 8). The Longitudinal Orthokeratology Research in Children (LORIC) study confirmed that OK lenses are an effective myopia control strategy (9). Recent studies have provided evidence that OK lenses could reduce axial elongation by 43%–63% over two years (10, 11). To explore the greater reduction in the progression of myopia, researchers looked at low concentrations of atropine combined with OK lenses as a therapy for myopia.

Several studies have explored the efficacy of the combination of 0.01% atropine and OK lenses in myopia control. The results showed that the combination of 0.01% atropine and OK lenses is more effective in controlling axial elongation than using OK lenses alone for one year or in the first few months (12–15). To our knowledge, no multicentre, double-blinded and placebocontrolled clinical trial has investigated the long-term effects of the combination of 0.01% atropine and OK lenses. Furthermore, it is worth noting the potential side effects and risks associated with combination therapy.

Previous studies have reported that atropine eyedrops can be used to make dry eye animal models (16). Therefore, when considering the long-term use of atropine in children, safety is as important as efficacy. It is necessary to be cautious about the possible influence of atropine on their ocular surfaces, especially on the tear film and meibomian gland. Therefore, we conducted a multicentre, randomized, double-blinded, placebo-controlled trial to investigate the efficacy and safety of the combination of 0.01% atropine and OK lenses on the progression of myopia in Chinese school-aged children.

## 2 Methods

### 2.1 Study design

This study was a randomized, placebo-controlled, double-blinded multicentre trial (registration No: ChiCTR1800018419. Registered on 17 September 2018). Ethical approval was obtained from the Institutional Review Board of Shanghai General Hospital [approval number: (2018)16], Wenzhou Medical University (approval number: Y2018-057), and Zhongshan Ophthalmic Center, Sun Yat-sen University (approval number: 2019KYPJ098). Written informed consent was obtained from both participating children and their parents or guardians. Children were eligible to participate in the study if they were 8–12 years of age at the time of consent, with myopia between −1.00 D and −4.00 D in either eye and astigmatism of no more than 1.5 D in either eye. The best-corrected visual acuity (BCVA) was no worse than 25/25 in both eyes. Participants with ocular disease (e.g., amblyopia, strabismus, allergic conjunctivitis and dry eye), those who had previously used OK lenses, atropine or other optical methods for myopia management, and those who had systemic morbidities that could influence refractive development or had difficulties completing follow-up measurements were excluded. The detailed study design has been reported previously (17).

### 2.2 Randomization

An independent statistician performed randomization of the 0.01% atropine or placebo eyedrop by using a web-based randomization program. To ensure balance in the site, a stratified and block randomization algorithm was adopted, randomization was stratified by the site, and block sizes of four were used within each stratum. The 0.01% atropine and placebo eyedrop were prepared in a manner that appeared similar in appearance (Shenyang Xingqi Pharmaceutical Co., China). Study medications were dispensed by designated coordinators who were not involved in outcome assessment. The masked examiners were responsible for the ophthalmic measurements. OK lens fitting was conducted by the same experienced optometrist in each study center. During the clinical trials, the study investigators, as well as the children and their guardians, were masked throughout the study.

### 2.3 Intervention

**Figure 1:**
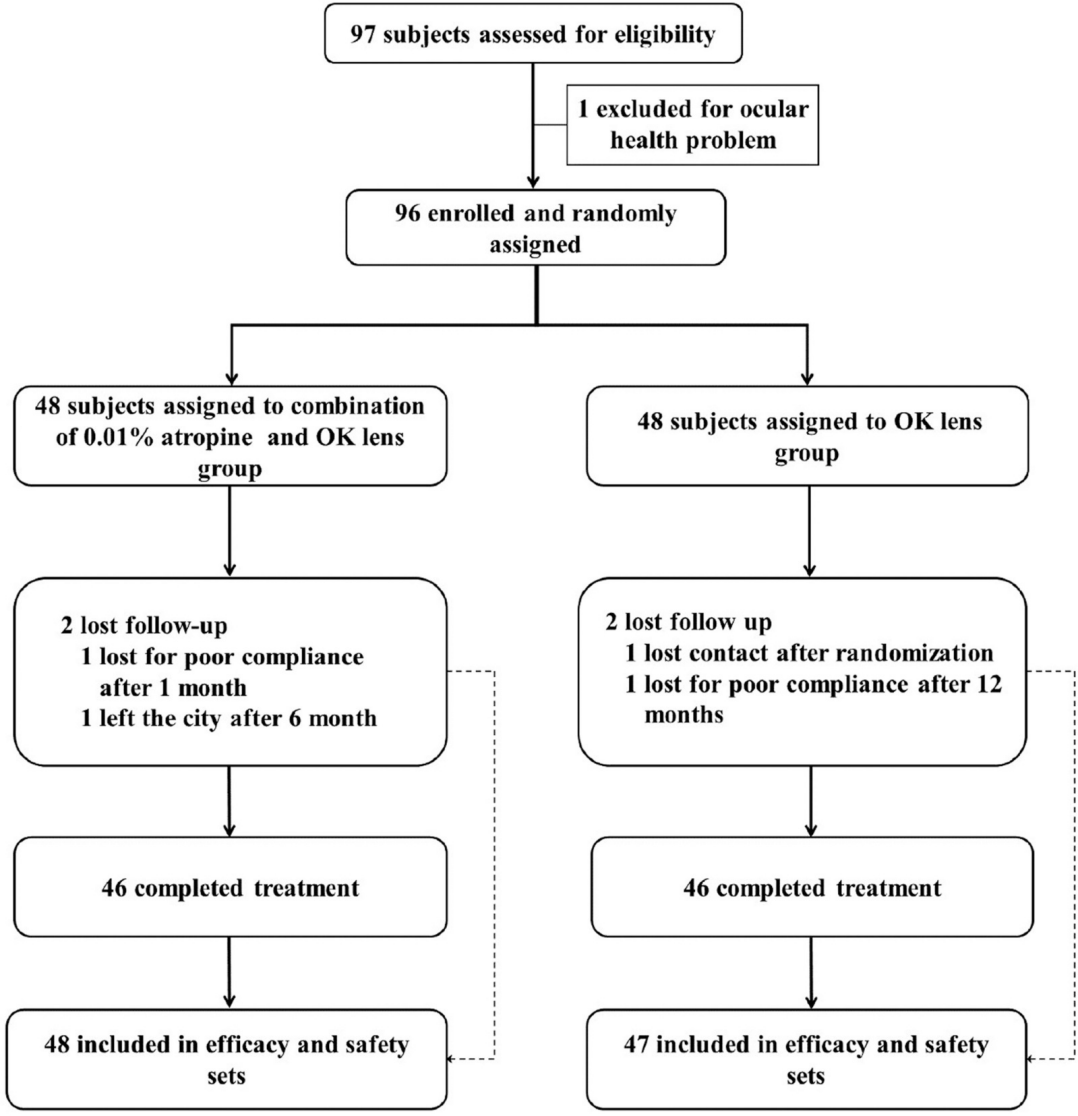
Subject recruitment and randomization flowchart.

Eligible participants were randomly assigned to the combination of 0.01% atropine and OK lens (AOK) group or the OK lens group at a 1:1 ratio. Subjects in the OK lens group used the OK lenses and the placebo eyedrop. Subjects in the AOK group used the OK lenses and 0.01% atropine. The OK lenses used in this study were Euclid lenses with a vision shaping treatment design. All subjects were instructed to wear OK lenses for at least 8 h every night. Before 10 min of wearing OK lenses, one drop of 0.01% atropine or placebo eyedrop was instilled. Participants were followed up at 1, 6, 12, 18, and 24 months after randomization. The study flow schedule is shown in Figure 1.

### 2.4 Outcome measurement

The primary outcome was the axial length (AXL) changes after 24 months. The secondary outcomes include the following: changes in first noninvasive break-up time (NIBUT), tear meniscus height (TMH), near visual acuity, distant visual acuity, corneal endothelial cell density, and adverse events at 24 months. During each follow-up visit, subjects and parents were asked about and recorded their medical illness or side effects, regardless of whether they appeared relevant to atropine or OK lenses. The dynamic changes of pupillary light reflex were also measured, including photopic and scotopic pupil size, constriction velocity and latency.

### 2.5 Measurements

All participants received comprehensive ophthalmological examinations at baseline and each follow-up visit. Refractive error was determined through comprehensive cycloplegic refraction. The corneal topography and AXL were measured using Pentacam (Oculus GmbH, Wetzlar, Germany). Corneal topography was used to confirm whether the decrease in visual acuity was due to decentration of the OK lenses. AXL was performed five times, and the average was taken. Corneal endothelial cell density was assessed using corneal endothelial microscopy (Tomey Corporation, Nagoya, Japan). TMH and NIBUT were obtained using the Keratograph 5M (Oculus GmbH, Wetzlar, Germany). Distant visual acuity was assessed using the Early Treatment Diabetic Retinopathy study (ETDRS) chart. Near visual acuity was obtained using a reduced ETDRS chart at 40 cm. Pupil measurements were taken with a NeurOptics PLR-300 pupillometer (NeurOptics, Irvine, CA), a handheld, monocular, infrared video pupillometer. After five minutes of dark adaptation, the measurements were taken in a completely dark room with the pupillometer as the solitary light source. The pupil measurements were taken under scotopic (ambient light intensity 0 uw) and photopic (ambient light intensity 50 uw) illumination conditions.

### 2.6 Statistical analysis

There was no follow-up result for two years at the beginning of this study. In a previous study (13), the AXL change was 0.09 ± 0.12 mm in the AOK group and 0.19 ± 0.15 mm in the OK lens group at the first year. Considering more power, the parameters of this new trial were conservatively set to be the same as those in previous studies, although the follow-up period was different. Based on this assumption and for a power of 80% and a 5% significance level, the sample size was 48 subjects per group, with an estimated dropout rate of 20%.

The subjects who took eyedrops and wore OK lenses at least once after randomization and had corresponding efficacy evaluations were analysed as the full analysis set (FAS). In the primary analysis, missing values in FAS will not be imputed, but multiple imputation will be used to deal with missing data in the sensitivity analysis, which aligns with the intention-to-treat principle. The per protocol set (PPS) includes the subjects in FAS who had good compliance [75% adherence rate (i.e., a mean of 5.25 days/week)] and complete efficacy evaluation. The primary outcome was analysed in the FAS and PPS, and secondary outcomes were analysed only in the FAS.

The statistical analysis was performed using SAS V.9.4 statistical software. If both eyes met the eligibility criteria, the left eye was regarded as the study eye. Continuous data are presented as the mean ± standard deviation (SD) or median (P25–P75), while categorical data are presented as numbers (percentages). For the primary outcome, a mixed-effect model adjusted for group, visits, interaction between group and visits, baseline level, age, and sex were used to compare the difference in change from baseline between the two groups and calculate its 95% CI. The statistical analysis strategy of secondary outcomes, including the NIBUT, TMH, distance and near visual acuity and dynamic changes of pupillary light reflex were the same as that of the primary outcomes. The distribution of adverse events of the two groups is described, and the chisquare test or Fisher's exact probability test was employed for comparison of incidence between the two groups. For the exploratory outcomes, the subgroup analysis in this trial was performed for age (≥8 & <10 years; ≥10 & ≤12 years) and SER (−1.0 D to −3.0 D; −3.0 D to −6.0 D). Statistical significance was set as two-sided *p* < 0.05 unless otherwise noted.

## 3 Results

Ninety-six subjects aged between 8 and 12 were enrolled in this study. Of those, 48 were allocated into the AOK group and 48 into the OK group (Figure 1). Since one subject in the OK group withdrew from the study without any follow-up visit after randomization. The FAS ultimately consisted of 95 subjects. Meanwhile, 83 subjects (86.5%) who completed the 24-month treatments were involved in the PP analysis, with 42 and 41 subjects in the AOK and OK groups, respectively. The characteristics of the subjects at baseline are shown in Table 1. No significant differences in age and sex distribution between the two groups. SER, AXL, NIBUT, TMH, and near and distant visual acuity were found no significant differences between the two groups of subjects at the baseline.

**Table 1:** Characteristics at baseline of subjects in the AOK group and the OK group.

	AOK	OK lens	*p* value
No. of subjects	48	47	
Age (years)	9.44 ± 1.11	9.60 ± 1.17	0.477
Sex (Male/Female)	17/31	19/28	0.615
Distance visual acuity	0.30 ± 0.18	0.32 ± 0.22	1.000
Near visual acuity	0.87 ± 0.15	0.86 ± 0.16	0.787
Degree of myopia (D)	−2.16 ± 0.90	−2.13 ± 0.86	0.896
Degree of astigmatism (D)	−0.50 ± 0.43	−0.38 ± 0.44	0.113
AXL (mm) (1 months)	24.54 ± 0.74	24.51 ± 0.68	0.843
NIBUT (s)	9.47 ± 3.99	8.56 ± 4.13	0.273
TMH (mm)	0.30 ± 0.21	0.31 ± 0.20	0.804
Corneal endothelial cell density	3,013.80 ± 236.61	3,078.34 ± 238.88	0.189

AOK, combined 0.01% atropine and orthokeratology lenses; OK lens, orthokeratology lens; AXL, axial length; NIBUT, non-invasive break-up time, TMH, tear meniscus height; Data are presented as mean ± SD.

### 3.1 Axial elongation

According to the FAS analysis, the mean AXL change from baseline was 0.33 mm (95% CI: 0.28–0.38) in the AOK group, which was significantly lower than 0.43 mm (95% CI: 0.38–0.47) in the OK group over the 24-month period (*p* < 0.01, Table 2). The mean axial elongation in the AOK group was 0.10 mm less than that in the OK group over 24 months (*p* < 0.01, Table 2). A time-dependent response to AXL control was observed in both groups. The AXL changes were significantly different between the two groups at visits of 18 months (*p* = 0.045). However, there were no significant changes between the two groups at visits of 6 and 12 months (*p* = 0.12, *p* = 0.20). The results of AXL change in the FAS analysis were supported by PP analysis (Table 2). In addition, the AXL change at 24 months was also similar and was statistically significant when missing data were imputed with multiple imputation (*p* = 0.031, Table 2).

**Table 2:** AXL changes (mm) in 2 years in the AOK group and the OK group.

Data set/followup visits	AOK (95%CI)	OK lens (95%CI)	Difference(95%CI)	*p* value
FAS set				
No. of patients	48	47		
6 months	0.05 (0.004 to 0.10)	0.10 (0.06 to 0.15)	−0.05 (−0.11 to 0.01)	0.124
12 months	0.16 (0.11 to 0.20)	0.20 (0.15 to 0.25)	−0.04 (−0.11 to 0.02)	0.201
18 months	0.25 (0.21 to 0.30)	0.32 (0.27 to 0.36)	−0.07 (−0.13 to −0.002)	0.045
24 months	0.33 (0.28 to 0.38)	0.43 (0.38 to 0.47)	−0.10 (−0.16 to −0.03)	0.004
PP set				
No. of patients	48	47		
6 months	0.06 (0.01 to 0.11)	0.10 (0.05 to 0.15)	−0.05 (−0.12 to 0.02)	0.195
12 months	0.16 (0.11 to 0.20)	0.20 (0.15 to 0.25)	−0.04 (−0.11 to 0.02)	0.207
18 months	0.24 (0.19 to 0.29)	0.31 (0.26 to 0.36)	−0.07 (−0.14 to −0.002)	0.045
24 months	0.32 (0.27 to 0.37)	0.42 (0.37 to 0.47)	−0.10 (−0.17 to −0.03)	0.006
Multiple Imputation (based FAS set, sensitivity analysis)				
6 months	−0.04 (−0.09 to 0.003)	0.070		
12 months	−0.04 (−0.09 to 0.01)	0.141		
18 months	−0.07 (−0.14 to −0.003)	0.061		
24 months	−0.10 (−0.18 to −0.009)	0.031		

AXL, axial length; AOK, combined 0.01% atropine and orthokeratology lenses; OK lens, orthokeratology lens; CI, confidence interval; FAS, full analysis; PP, per protocol; Data are presented as mean and its 95% CI.

**Figure 2:**
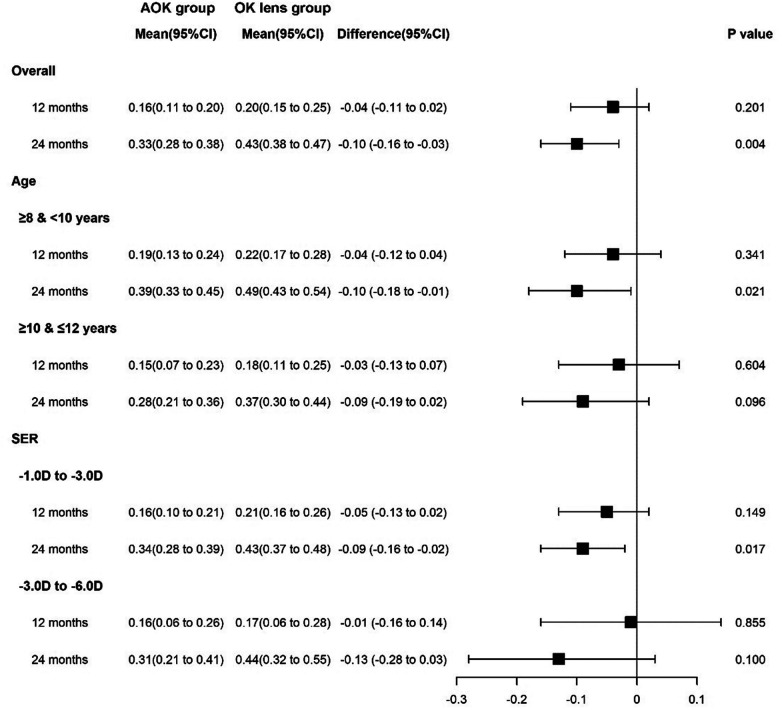
Forest plot of AXL at 12- and 24-month visit in the subgroup of SER and age. AOK, combined 0.01% atropine and orthokeratology lenses; OK lens, orthokeratology lens; CI, confidence interval; SER, spherical equivalent refraction; Data are presented as mean and its 95% CI.

Next, we stratified the subjects by age and SER at enrollment. In the subgroup of subjects aged 8.0–9.9 years (25 subjects in the AOK group and 23 in the OK group), only changes in AXL at the 24-month visit were significantly smaller in the AOK group than in the OK group (*p* = 0.021, Figure 2). In contrast, in the subgroup of subjects aged 10.0–12.0 years (23 subjects in the AOK group and 24 in the OK group), the changes in AXL did not differ significantly between the AOK group and the OK group over 12- and 24month periods (*p* = 0.604, 0.096, respectively, Figure 2).

In the subgroup of subjects with an SER of −1.00 to −3.00 D (35 subjects in the AOK group and 37 in the OK group), only changes in AXL at the 24-month visit were significantly smaller in the AOK group than in the OK group (*p* = 0.017, Figure 2). In contrast, in the subgroup of subjects with an SER of −3.01 to −6.00 D (13 subjects in the AOK group and 10 in the OK group), changes in AXL over 12- and 24-month periods were insignificantly smaller in the AOK group than in the OK group (*p* = 0.855, 0.100, respectively, Figure 2).

### 3.2 Change in TMH, NIBUT, distant and near visual acuity and dynamic changes of pupillary light reflex

After 24 months of treatment, the change in TMH was 0.01 (95% CI: −0.01 to 0.04) and 0.03 (95% CI: 0.004–0.05) in the AOK and OK groups, respectively. No statistically significant difference was found in the TMH changes between the AOK and OK groups (*p* = 0.344) (Supplementary Figure S1). The changes in NIBUT were −0.46 (95% CI: −1.42 to 0.50) and 0.66 (95% CI: −0.28 to 1.60) in the AOK and OK groups, respectively. The change of NIBUT had no significant difference between the two groups (*p* = 0.103). There were no significant differences in distant and near visual acuity between the two groups (*p* > 0.05, all) (Supplementary Figure S1).

Photopic pupil size was significantly increased after 24 months of treatment (*p* < 0.01, respectively) in the AOK group, while the scotopic pupil size was not significantly changed (*p* > 0.05) (Supplementary Figure S1). In the OK group, both the photopic and scotopic pupil size was not significantly changed (p>0.05, all) (Supplementary Figure S1). Significant differences in constriction velocity changes between the two groups were only observed at the 12-month visit (*p* = 0.033) (Supplementary Figure S2). The latency of the pupillary light reflex in both eyes in the AOK group was significantly prolonged over 18- and 24-month periods (*p* = 0.032, 0.015, respectively) (Supplementary Figure S2). There was a significant difference in the latency compared with the OK group at the same time points (*p* = 0.011, 0.029, respectively) (Supplementary Figure S2).

### 3.3 Adverse events

A summary of adverse events is provided in Table 3. No severe complications, such as infectious keratitis, were observed. Three subjects in the AOK group and four subjects in the OK group had mild corneal staining. Their mild corneal staining resolved approximately 1 week after discontinuation of the OK lenses. Three subjects in the AOK group and four subjects in the OK group developed conjunctivitis and thus suspended treatment. After topical antimicrobial therapy for one week, the conjunctivitis was resolved. One subject in the AOK group developed meibomianitis after 6 months of treatment and suspended treatment for one month. One subject in the AOK group felt sensitive to light after awakening in the morning, which lasted for approximately half an hour at the 1-month visit. However, there was no significant disturbance to the daily activities. No other adverse events were reported.

**Table 3:** Summary of adverse events over 24 months.

Adverse events	Severity	AOK group (*n* = 48) *n* (%)	OK lens group (*n* = 47) *n* (%)
Corneal staining	Mild	3 (6.25%)	4 (8.51%)
Conjunctivitis	Moderate	3 (6.25%)	4 (8.51%)
Meibomianitis	Moderate	1 (2.08%)	0
Photophobia	Mild	1 (2.08%)	0

## 4 Discussion

Previous studies have reported that myopia progression in school-age children is slowed by the combination treatment of 0.01% atropine and OK lens. To clarify the long-term efficacy and safety, further evidence is still needed. Our findings revealed that, based on the data of AXL changes, a 24-month treatment with AOK reduced myopia progression. Furthermore, minimal side effects were observed with the combination treatment.

The mean axial elongation in previous randomized trials was shown in Table 4 that included the use of OK lens or the combination of 0.01% atropine and OK lens. In the current study, AOK treatment achieved better efficacy than monotherapy with OK lenses in 2 years, which was consistent with a previous study (12, 15). The AXL increased by 0.33 mm in the AOK treatment over 2 years, which was similar to that of the previous study of Yang et al. (0.31 ± 0.23). Meanwhile, the 2-year AXL increased by 0.43 mm in the monotherapy OK lens group. This was also consistent with the previous result of Yang et al. (0.43 ± 0.22). However, the 2-year AXL elongation in both groups was slightly greater than that in previous studies by Kinoshita et al. Notably, the two studies vary in a few aspects. The mean age of subjects was 10.3 years old in Kinoshita et al.'s study compared with 9.52 years old in the current study. The refraction was −2.7 D in Kinoshita et al.'s study, which was higher than the refraction (−2.1 D, range from −1.00 to −4.00 D) in this study. As already noted, younger age was associated with a higher AXL elongation rate (18, 19). In this study, axial elongation was greater in the subgroup aged 8–10 years than in the subgroup aged 10–12 years in both the AOK and OK groups. This may be the reason why the combination treatment group showed more AXL elongation when compared to previous studies (14, 20). Notably, the additional benefit of combination therapy appeared to emerge over time, with significant differences observed only at 18 and 24 months, suggesting a cumulative treatment effect. Regarding potential mechanisms, our findings on pupillary dynamics provide further insight. The increases in photopic pupil size and prolonged light reflex latency observed in the AOK group at 18- and 24-month visits aligned with its greater efficacy in slowing axial elongation. These later pupillary changes may contribute to enhanced control, potentially through increased light exposure and amplified peripheral myopic defocus (21, 22). Future studies are required to establish the definitive mechanism of the combined effect of combining 0.01% atropine and OK lens.

**Table 4:** Previous RCTs related to the efficacy of 0.01% atropine drops, OK lens or AOK treatment.

	Sample size (n)	Baseline age	Baseline SER(D)	1-year AL change (mm)		2-year AL change (mm)	
				OK	AOK	OK	AOK
Tan et al	48 AOK and 48 OK	6–11	−1.00 to −4.00	0.16 ± 0.15	0.07 ± 0.16	0.34 ± 0.03	0.17 ± 0.03
Kinoshita et al	40 AOK and 40 OK	8–12	−1.00 to −6.00	0.19 ± 0.15	0.09 ± 0.12	0.40 ± 0.23	0.29 ± 0.20
Zhao and Hao	20 AOK and 20 OK	5–14	−1.00 to −6.00	0.29 ± 0.11	0.14 ± 0.08		
Xu and Yang	42 AOK and 40 OK	8–12	−1.00 to −6.00	0.24 ± 0.17	0.20 ± 0.23	0.43 ± 0.22	0.31 ± 0.23
Current study	48 AOK and 47 OK	8–12	−1.00 to −6.00	0.20 ± 0.16	0.16 ± 0.14	0.43 ± 0.16	0.33 ± 0.17

SER, spherical equivalent refraction; AXL, axial length; AOK, combined 0.01% atropine and orthokeratology lenses; OK lens, orthokeratology lens; Data are presented as mean ± SD.

As the effect of refraction and age had an important role in the efficacy of the OK lens and atropine, we further stratified the subjects by the baseline SER and age, and the difference in AXL elongation between the AOK and OK groups was found to be significant in subjects with a SER of −1.00 to −3.00 D or aged 8–10 years. In contrast, there was no significant difference between the two groups in subjects with a SER of −3.00 to −6.00 D or aged 10–12 years. One possible explanation is that children in the higher myopia subgroup were slightly older and may have had slower physiological progression. However, baseline age was included as a covariate in our models, suggesting that the SER-dependent differences are unlikely to be explained by age alone. A more plausible explanation is OK lenses alone may already provide substantial control in children with higher myopia. Previous studies have shown that lower baseline SER and younger age are associated with a better response to low-dose atropine, whereas OK lenses tend to show stronger efficacy in moderate to higher myopia (9, 15, 23–27). Importantly, as the first multicenter, randomized, double-blinded clinical trial conducted across different cities in China, our study provides higher-level evidence to evaluate the combined efficacy of combined 0.01% atropine and OK for slowing axial elongation in children.

In this study, we were also cautious about the possible influence of combined atropine and OK lenses on ocular surfaces. It was reported that atropine may have had an inhibitory effect on tear secretion, as it may act on the muscarinic receptors in the lacrimal gland, which causes a significant decrease in aqueous production (28). A previous study suggested that 0.01% atropine may have no influence on Schirmer's test in the first year (20). However, long-term evidence is lacking. Based on our results, there was no significant difference between the AOK and OK groups for TMH over 2 years. Additionally, the TMH did not have a declining tendency in either the AOK or OK groups after 24 months of treatment compared with enrolment. Moreover, considering the potential influence on the stability of the tear film, our results showed no significant difference between the AOK and OK groups for NIBUT over 2 years. When compared with enrolment, we observed a slight decline in NIBUT in the AOK group but not in the OK group. This was indicated by a previous preliminary study. They mentioned that some children with long-term atropine treatment may have abnormal morphology in tear film (29). Therefore, the combination of 0.01% atropine and OK lenses has little effect on tear secretion and tear film stability.

Notably, this study had several limitations. Firstly, our study did not have a control group using 0.01% atropine alone. Although 0.01% atropine alone can slow myopia progression, atropine ophthalmic solution treatment must be combined with spectacles to provide refractive correction. The study was originally designed to be a double-blinded clinical trial. It would not be reasonable to use 0.01% atropine alone. In addition, the current study explored the additive effect of combining 0.01% atropine with an OK lens, using an OK lens alone as a comparator. The absence of an atropine-only group did not affect the investigation of a combined treatment. Secondly, our study did not measure peripheral refraction or higher-order aberrations, which may clarify the mechanism of the additive effects of 0.01% atropine and OK lenses.

In conclusion, the current multicentre RCT study confirmed the long-term efficacy of combined 0.01% atropine and OK lens treatment in children aged 8–12 years old. Combined treatment can significantly improve the efficacy compared with monotherapy with OK lenses. The combined treatment was well accepted with negligible influence on the ocular surface.

## Data Availability

The original contributions presented in the study are included in the article/Supplementary Material, further inquiries can be directed to the corresponding authors.
