# Supplementary material for: The efficacy and safety of combined 0.01% atropine and orthokeratology for childhood myopia control: a 2-year randomized clinical trial
Source: Front Pediatr. 2026 Apr 30;14:1809296. doi: 10.3389/fped.2026.1809296 (PMC13171514; doi:10.3389/fped.2026.1809296)
Supplement: Supplementary file 1 [file Datasheet1.pdf]

**The efficacy and safety of combined 0.01% atropine and orthokeratology for childhood myopia control: A 2-year randomized clinical trial**

Ying Yuan[1,3#], Yuqi Deng[2#], Chengcheng Zhu[4], Xinting Liu[5], Wei Zhang[6], Bingru Zheng[7], Xinjie Mao[5*], Xiao Yang[8*], Bilian Ke[2*]

1. Department of Ophthalmology, Shanghai General Hospital, Shanghai Jiao Tong University School of Medicine; Shanghai, China;
2. Department of Ophthalmology, Renji Hospital, Shanghai Jiao Tong University School of Medicine, Shanghai, China
3. National Clinical Research Center for Eye Diseases; Shanghai, China;
4. Qilu Hospital of Shandong University Dezhou Hospital, Dezhou, Shandong Province, China;
5. National Clinical Research Center for Ocular Diseases, Eye Hospital, Wenzhou Medical University, Wenzhou, China;
6. Biomedical informatics & statistics center, School of Public Health, Fudan University, Shanghai, China;
7. Shenzhen Eye Hospital, Jinan University, Shenzhen Eye Institute, Shenzhen, China;
8. State Key Laboratory of Ophthalmology, Zhongshan Ophthalmic Center Sun Yat-Sen University, Guangzhou, China;

[#] Ying Yuan and Yuqi Deng contributed equally to this work

* Xinjie Mao, Xiao Yang and Bilian Ke are co-correpsonding authors of this manuscript.

Corresponding author and address for reprint requests

*Bilian Ke

Department of Ophthalmology, Renji Hospital, Shanghai Jiao Tong University School of Medicine, Shanghai, China, Shanghai 200127, 021-58752345， China

E-mail: kebilian@126.com

*Xinjie Mao

National Clinical Research Center for Ocular Diseases, Eye Hospital, Wenzhou Medical University, Wenzhou 325027, 0577-88068888, China

E-mail: mxj@mail.eye.ac.cn

*Xiao Yang

Sun Yat-Sen University Zhongshan Ophthalmic Center State Key Laboratory of Ophthalmology, Guangzhou, Guangdong 510000, 020-66607666, China

E-mail: Yangx_zoc@163.com

**Supplementary files**

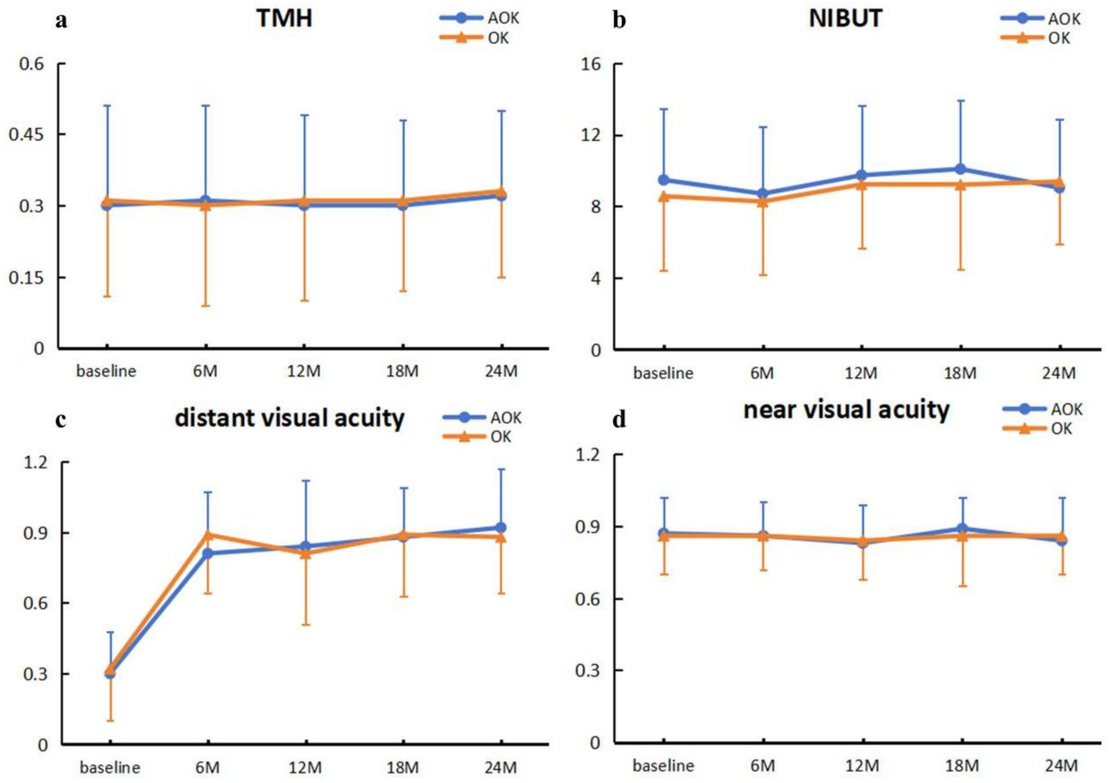


**Figure S1. The TMH, NIBUT, distant visual acuity and near visual acuity at enrolment and each follow-up visits.**

a. the TMH at enrolment, 1 months, 6 months, 12 months, 18 months and 24 months; b. the NIBUT at enrolment, 1 months, 6 months, 12 months, 18 months and 24 months; c. distant visual acuity at enrolment, 1 months, 6 months, 12 months, 18 months and 24 months; d. near visual acuity at enrolment, 1 months, 6 months, 12 months, 18 months and 24 months.

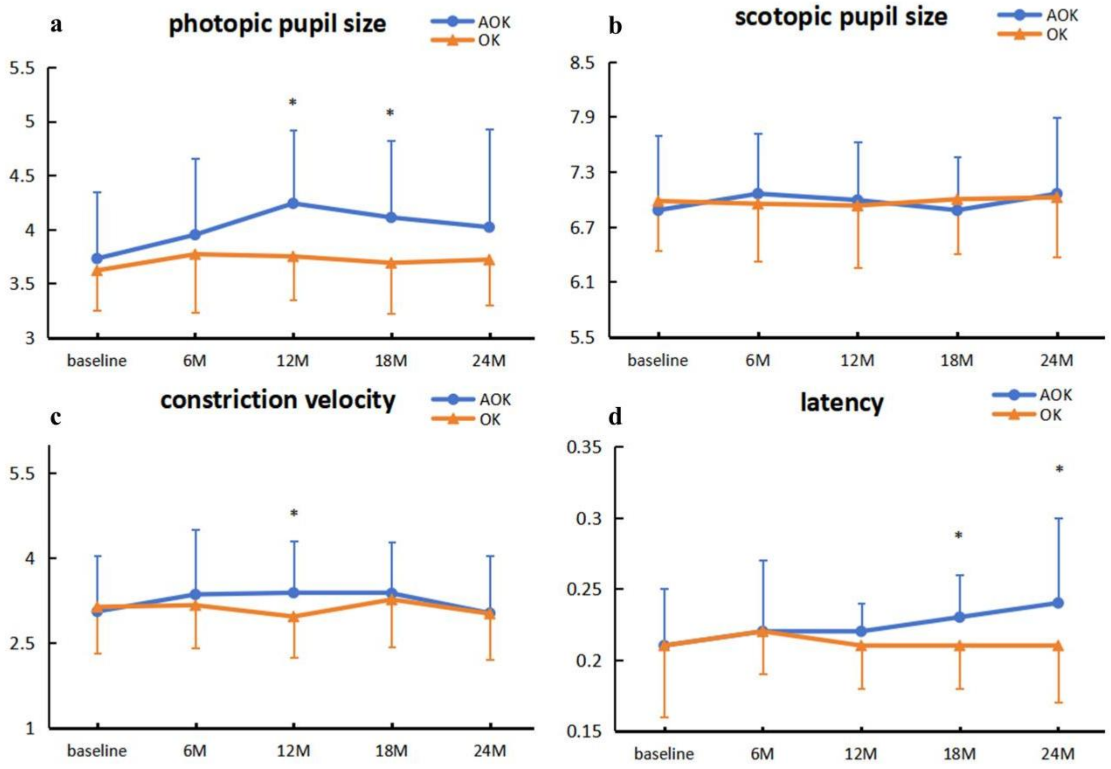


**Figure S2. The photopic pupil size, scotopic pupil size, constriction velocity and latency of pupillary light reflex at enrolment and each follow-up visits.**

a. the photopic pupil size at enrolment, 1 months, 6 months, 12 months, 18 months and 24 months; b. the scotopic pupil size at enrolment, 1 months, 6 months, 12 months, 18 months and 24 months; c. the constriction velocity at enrolment, 1 months, 6 months, 12 months, 18 months and 24 months; d. latency of pupillary light reflex at enrolment, 1 months, 6 months, 12 months, 18 months and 24 months.

*, P $<0.05$, compared with OK group.
